# Curvature Induced by Deflection in Thick Meta‐Plates

**DOI:** 10.1002/adma.202008082

**Published:** 2021-06-13

**Authors:** Mohammad J. Mirzaali, Aref Ghorbani, Kenichi Nakatani, Mahdiyeh Nouri‐Goushki, Nazli Tümer, Sebastien J. P. Callens, Shahram Janbaz, Angelo Accardo, José Bico, Mehdi Habibi, Amir A. Zadpoor

**Affiliations:** ^1^ Department of Biomechanical Engineering Faculty of Mechanical Maritime and Materials Engineering Delft University of Technology (TU Delft) Mekelweg 2 Delft 2628 CD The Netherlands; ^2^ Physics and Physical Chemistry of Foods Department of Agrotechnology and Food Sciences Wageningen University Wageningen 6708 WG The Netherlands; ^3^ Department of Precision and Microsystems Engineering Delft University of Technology Mekelweg 2 Delft 2628 CD The Netherlands; ^4^ Sorbonne Université Université Paris Diderot and Laboratoire de Physique et de Mécanique des Milieux Hétérogenes (PMMH) CNRS ESPCI Paris PSL Research University – 10 rue Vauquelin Paris 75005 France

**Keywords:** 3D printing, auxeticity, functional materials, Gaussian curvature, mechanical metamaterials

## Abstract

The design of advanced functional devices often requires the use of intrinsically curved geometries that belong to the realm of non‐Euclidean geometry and remain a challenge for traditional engineering approaches. Here, it is shown how the simple deflection of thick meta‐plates based on hexagonal cellular mesostructures can be used to achieve a wide range of intrinsic (i.e., Gaussian) curvatures, including dome‐like and saddle‐like shapes. Depending on the unit cell structure, non‐auxetic (i.e., positive Poisson ratio) or auxetic (i.e., negative Poisson ratio) plates can be obtained, leading to a negative or positive value of the Gaussian curvature upon bending, respectively. It is found that bending such meta‐plates along their longitudinal direction induces a curvature along their transverse direction. Experimentally and numerically, it is shown how the amplitude of this induced curvature is related to the longitudinal bending and the geometry of the meta‐plate. The approach proposed here constitutes a general route for the rational design of advanced functional devices with intrinsically curved geometries. To demonstrate the merits of this approach, a scaling relationship is presented, and its validity is demonstrated by applying it to 3D‐printed microscale meta‐plates. Several applications for adaptive optical devices with adjustable focal length and soft wearable robotics are presented.

## Graphical abstract

The design of advanced functional devices often requires intrinsically curved geometries that belong to the realm of non‐Euclidean geometry. Thick meta‐plates based on hexagonal cellular mesostructures are used to achieve a wide range of intrinsic (i.e., Gaussian) curvatures. A scaling relationship is presented to predict the deformation‐induced curvature of thick plates and their micro‐architectural design variables.

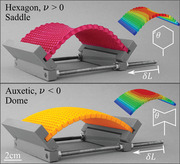

## 1 Introduction

Curved surfaces are ubiquitous in nature. Biology, in particular, thrives on curved objects, as a growing body of recent evidence suggests.^[^ ^1^, ^2^, ^3^ ^]^ Fundamental processes, such as cell migration,^[^ ^4^, ^5^ ^]^ morphogenesis,^[^ ^6^, ^7^ ^]^ and tissue regeneration^[^ ^8^, ^9^ ^]^ are often dependent on the curvature of their surrounding environments.^[^ ^10^ ^]^ The design of dynamic and programmable curved surfaces, however, remains challenging for engineers. These challenges can, for example, be observed in the case of thin‐walled structural elements, which are very popular due to their combination of lightness, load transfer efficiency,^[^ ^11^, ^12^, ^13^, ^14^ ^]^ and low cost. Although an initially flat panel can be reasonably bent in a single direction to adapt the shape of an arch, transforming the panel into a shell dome or a saddle and, thus, changing its Gaussian curvature remains challenging.^[^ ^15^, ^16^, ^17^, ^18^ ^]^ Several studies have used computer‐aided design using conformal geometry,^[^ ^19^, ^20^ ^]^ origami,^[^ ^21^, ^22^, ^23^, ^24^, ^25^, ^26^, ^27^, ^28^, ^29^, ^30^ ^]^ kirigami,^[^ ^31^, ^32^ ^]^ or crumpling^[^ ^33^, ^34^ ^]^ approaches to create curved objects from flat sheets. However, these approaches rely on very thin sheets and only lead to an approximation of the desired curvature. Other techniques based on non‐uniform swelling^[^ ^35^, ^36^ ^]^ or inflation^[^ ^37^ ^]^ or liquid crystal phase transition^[^ ^38^ ^]^ have also been proposed, but these structures are made of soft materials and may not be suitable for large‐scale and/or load‐bearing structures. “Bending‐active system”^[^ ^39^, ^40^ ^]^ is another approach to create form‐finding structures. These form‐finding structures rely on the elastic deformation of a combination of several structural elements (e.g., vector‐active, surface‐active, form‐active, etc.) that are initially planar or straight.^[^ ^40^, ^41^, ^42^, ^43^, ^44^ ^]^ Therefore, individual curved beam, shell, or membrane elements of bending‐active systems remain elastically constrained and can carry residual bending stresses.^[^ ^40^ ^]^ Therefore, patterning individual elements and their pre‐stress condition can control the shape of the bent elements. This is why bending‐active design strategy is mostly considered as a suitable approach for building arbitrarily curved objects rather than continuous structures. This approach can also be used for the design of compliant mechanisms.^[^ ^40^ ^]^

Following Carl Friedrich Gauss's “Egregium” theorem, the only theoretically admissible way of obtaining intrinsically curved geometries from flat sheets is to allow for in‐plane deformations.^[^ ^21^, ^45^ ^]^ The physics of continuum plates has been extensively studied using classical elasticity theories. As an example, Kirchhoff–Love's classical theory of plates^[^ ^46^ ^]^ explains the mechanics of thin plates, where through‐the‐plane shear effects are eliminated, but the Reissner–Mindlin's theory describes the mechanics of thick plates by considering through‐the‐plane shear deformations.^[^ ^47^, ^48^, ^49^ ^]^ Some other examples of the approaches that have been applied to improve our current understanding of the mechanical behavior of curved structures are higher‐order shear‐deformation theory^[^ ^50^, ^51^ ^]^ and bending gradient theory^[^ ^52^ ^]^ that have been used to analyze the curvature of composites plates and differential geometry^[^ ^53^, ^54^, ^55^ ^]^ that can be utilized to study curved objects in the 3D Euclidean space. It is often challenging to derive exact solutions for these analytical problems. Therefore, finite element (FE)‐based (homogenization) approaches can be used to analyze the bending behavior of cellular structures.^[^ ^56^, ^57^, ^58^, ^59^ ^]^

Even though the mechanics of continuum plates have been well studied, “thick” non‐isotropic plates that exhibit auxeticity (i.e., a negative Poisson's ratio) have been less explored,^[^ ^60^, ^61^, ^62^ ^]^ particularly under bending deformations. We propose a combination of computational modeling and experiments to study how curvature develops in thick plates exhibiting auxetic or non‐auxetic behaviors as a result of mechanical deformation. One way to manipulate the Poisson's ratio of a plate is to use architected designs such as those found in cellular metamaterials. Rationally designing the small‐scale geometry of metamaterials allows for creating unusual macroscale properties, such as negative values of the Poisson's ratio^[^ ^61^, ^63^, ^64^, ^65^, ^66^, ^67^ ^]^ and stiffness,^[^ ^68^, ^69^ ^]^ as well as for shape adjustments, such as shape‐matching,^[^ ^70^ ^]^ shape morphing,^[^ ^71^, ^72^, ^73^ ^]^ shape‐shifting,^[^ ^19^ ^]^ or shape integrity.^[^ ^74^ ^]^ The prefix “meta” is sometimes also applied to structural elements with similar unusual behaviors (e.g., metabeams^[^ ^75^ ^]^). It is within this context that we refer to our thick plates with different distributions of the Poisson's ratio as “meta‐plates.”

**Figure 1:**
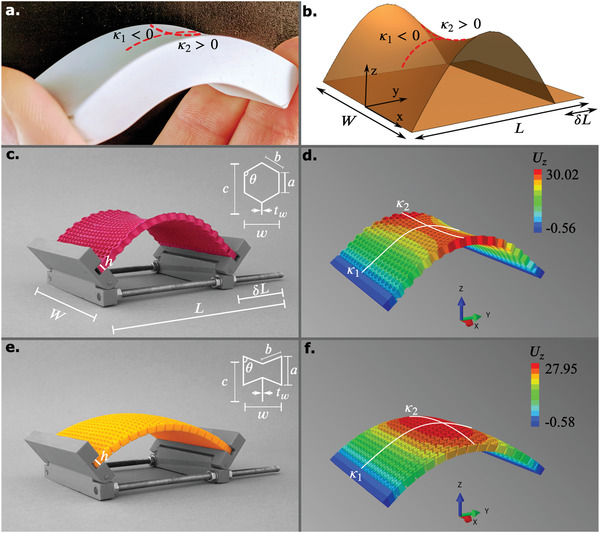
a) An example of an intrinsically curved shape with two non‐zero principal curvatures resulting from the bending of a rubber eraser. b) A sketch of the experimental setups and c–f) computational models of non‐auxetic (c,d) and auxetic (e,f) meta‐plates. The actual geometrical parameters used in the experiments and computations are presented in Table S1, Supporting Information. The numerical models depict (in contour lines) the vertical displacement, $U_{z}$, (mm).

Here, we focus on the Gaussian curvature that describes the intrinsic curvature of a surface that can be “felt” by the inhabitants of that surface. This is in contrast with the extrinsic curvature, which requires the embedding into a space of higher dimension to be observed. The Gaussian curvature of a surface, κ, is obtained as the product of its two principal curvatures, $\kappa_{1}$ and $\kappa_{2}$:$\kappa =\kappa_{1}.\kappa_{2}$ (**Figures** **1**a,b). Depending on the values of these principal curvatures, three types of surfaces, namely synclastic (i.e., dome‐like), monoclastic (i.e., zero‐curvature), or anticlastic (i.e., saddle‐like) can be defined.

## 2 Results and Discussion

Bending a rubber eraser along its length results in a curvature of opposite sign along the transverse direction (Figure 1a). The origin of such induced curvature is the Poisson's ratio, ν, which relates the strain observed along a transverse direction to the strain applied along the longitudinal direction. Usual materials have a positive Poisson's ratio and tend to get compressed in the transverse direction under uniaxial stretching. As the eraser is bent with a curvature $\kappa_{1}$, its upper part is stretched while the lower part is compressed, leading to opposite strains in the transverse direction. As a result, a curvature $\kappa_{2}=‐\nu \kappa_{1}$, appears in the transverse direction giving rise to a familiar saddle shape (Figures 1a,b). However, this anticlastic effect is only observed in the case of small‐width plates that approach the slender shape of a beam. Bending a wide plate generally results in a single curvature (i.e., zero Gaussian curvature), except for a fraction of the width of the plate, which is of the order ${\left(h/\kappa_{1}\right)}^{1/2}$, where *h* is the thickness of the plate.^[^ ^76^ ^]^ The absence of the Poisson‐induced curvature along the whole width is due to the additional cost in the stretching energy corresponding to the change of the Gaussian curvature. This paradigm is, however, challenged when considering cellular panels in which transverse curvatures are observed even for relatively large specimen widths.^[^ ^77^, ^78^ ^]^ This effect can be interpreted as a consequence of a relatively low in‐plane stretching stiffness of such meta‐plates, while the flexural rigidity remains high.^[^ ^79^ ^]^ Such combinations of in‐plane and flexural mechanical properties can be used to accommodate Gaussian curvatures in thick meta‐plates.

We designed thick cellular plates using hexagonal lattices characterized by an angle θ. Depending on the value of this angle, such meta‐plates display either a positive (θ>90°) or a negative (θ<90°) value of the Poisson's ratio, respectively leading to negative (Figures 1c,d) or positive (Figures 1e,f) Gaussian curvatures upon bending. Numerical simulations based on the FE method display the same features and will be used to study the impact of the different geometrical parameters on the induced transverse curvature.

Consider the out‐of‐plane buckling in the *Z*‐direction of a meta‐plate under compression in the Y‐direction, while hinges provide free rotation at the ends (Figures 1c–f). From the classical theory of buckling, we expect the vertical displacement of the plate to follow a sinusoidal function of the longitudinal coordinate with a half period equal to the length of the plate.^[^ ^80^, ^81^ ^]^ From symmetry, the displacement of the transverse coordinate should follow an even function. As a simplifying approximation, a quadratic function multiplied by the sinusoidal function can be used to predict the out‐of‐plane deformation of a thick meta‐plate:

(1) $$\begin{array}{c} w\left(x,y\right)=\left(A+Bx^{2}\right)\;\left(\sin\;Cy\right) \end{array}$$

where C=π/L and A and B are constants determined by fitting Equation (1) to our experimental data points. We deduce the main curvatures at the middle point of the surface $\kappa_{1}=‐AC^{2}$ and $\kappa_{2}=2B$. We expect $\kappa_{2}$ to vanish for small values of the meta‐plate thickness.

To estimate the Poisson's ratio of our structures, we used the existing theoretical relations based on rigid frames connected by hinges:^[^ ^82^ ^]^

(2) $$\begin{array}{c} \nu =\frac{-\cos\theta }{\left(a/b-\cos\theta \right)} \end{array}$$

where a and b are the lengths of the struts of the hexagonal lattices (Figures 1c,e). The values of vary between 48° and 120°. Although the actual values of ν should also depend on the applied strain, we assumed that ν does not change significantly during bending. The reported values of the Poisson's ratios, thus, pertain to the initial stage of the deformations. Beyond the Poisson's ratio, several other parameters characterize the metastructure, including the cell size, $w=2b\sin\left(\theta \right)$, the thickness of the cell wall, $t_{w}$, the thickness of the plate, h, and the width of the plate, W (Figure 1c). The applied load controls the longitudinal curvature, $\kappa_{1}$, through uniaxial compression. We used experiments and FE models to study how the transverse curvature, $\kappa_{2}$, varies as a function of these different parameters.

In the experiments and numerical simulations, our reference meta‐plate is based on an array of 15 × 18 hexagonal cells with the following dimensions, $w_{0}$ = 5.1 mm, c = 7.65 mm, $t_{w}$ = 0.51 mm, and W = 92 mm (Figure 1). Other specimens were designed based on multiples or fractions of these nominal parameters.

**Figure 2:**
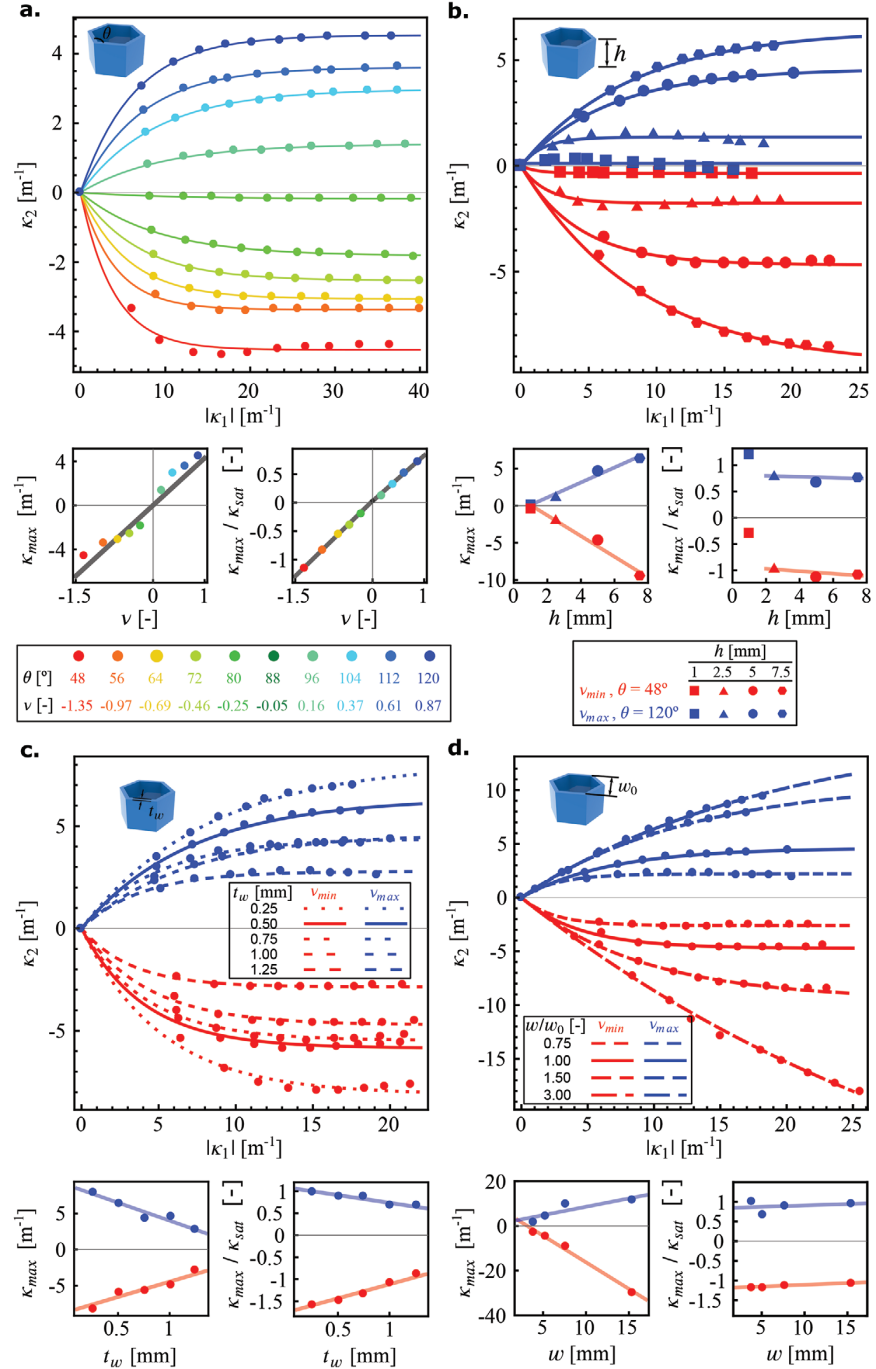
a–d) The evolution of the second principal curvature with respect to the first principal curvature for meta‐plates with different values of the Poisson's ratio (a), as well as different values of the thickness, h (b), unit cell thickness, $t_{w}$ (c), and unit cell width, w (d), in two extreme configurations (for each variation of a geometrical parameter, the other parameters were kept constant). The dependence is linear only for the lowest value of $\kappa_{1}.\kappa_{2}$, as in the classical case of a rubber eraser but tends to saturate for the higher values of $\kappa_{1}$. The variation of $\kappa_{2}$ with $\kappa_{1}$ can be described using an exponential function (i.e., $\kappa_{2}=\kappa_{max}\;\times \;\left[1-e^{\frac{-\kappa_{1}}{\kappa_{sat}}}\right]$). Insets show the evolution of the fitting parameters with the geometrical characteristics of the meta‐plates.

The specimens were uniaxially compressed in a stage. Beyond a buckling load, they adopt a longitudinal curvature $\kappa_{1}$ and a transverse curvature $\kappa_{2}$. As the load is increased, the out‐of‐plane buckling is amplified. Initially, $\kappa_{2}$ increases proportionally to $\kappa_{1}$, as in the case of a bent eraser, but then saturates (**Figure** **2**). This behavior can be approximately captured by a saturating exponential of the form $\kappa_{2}\;=\;\kappa_{\max}\;\times \;\left[1-e^{\frac{-\kappa_{1}}{\kappa_{\text{sat}}}}\right]$. For $\kappa_{1}\ll \kappa_{sat}$, we expect a linear evolution of the form $\kappa_{2}=\frac{\kappa_{\max}}{\kappa_{\text{sat}}}\kappa_{1}$. This motivates our interests in the prefactor $\frac{\kappa_{\max}}{\kappa_{\text{sat}}}$. We find that the maximum curvature ($\kappa_{max}$) reached by the meta‐plates is linearly correlated to its Poisson's ratio (Figure 2a, bottom‐left), thickness (Figure 2b, bottom‐left), as well as to the thickness (Figure 2c, bottom‐left) and width (Figure 2d, bottom‐left) of its unit cells. While the initial slope, $\frac{\kappa_{\max}}{\kappa_{\text{sat}}}$, varies linearly with the Poisson's ratio (Figure 2a, bottom‐right), it is approximately independent of the other geometrical parameters (Figures 2b–d, bottom‐right).

We also observe a decrease of $\kappa_{max}$ as the thickness of the walls increases (Figure 2c, bottom‐left). Increasing the thickness of the meta‐plates for the extreme values of the Poisson's ratio increases the rate of the evolution of the second principal curvature (Figure 2b). Increasing the thickness of the unit cell struts, $t_{w}$, affects the evolution of the second principal curvature as well (Figure 2c). We also evaluated the evolution of the curvature when the unit cell size was scaled. Toward that end, we changed the number of the unit cells while keeping their relative size constant (i.e., $\frac{c}{w}=\frac{3}{2}$). Meta‐plates with a higher number of overall unit cells (i.e., $\frac{w}{w_{0}}=3$) reach higher values of the second principal curvature (Figure 2d), and the rate of the evolution of the curvature for those upscaled structures is lower.

**Figure 3:**
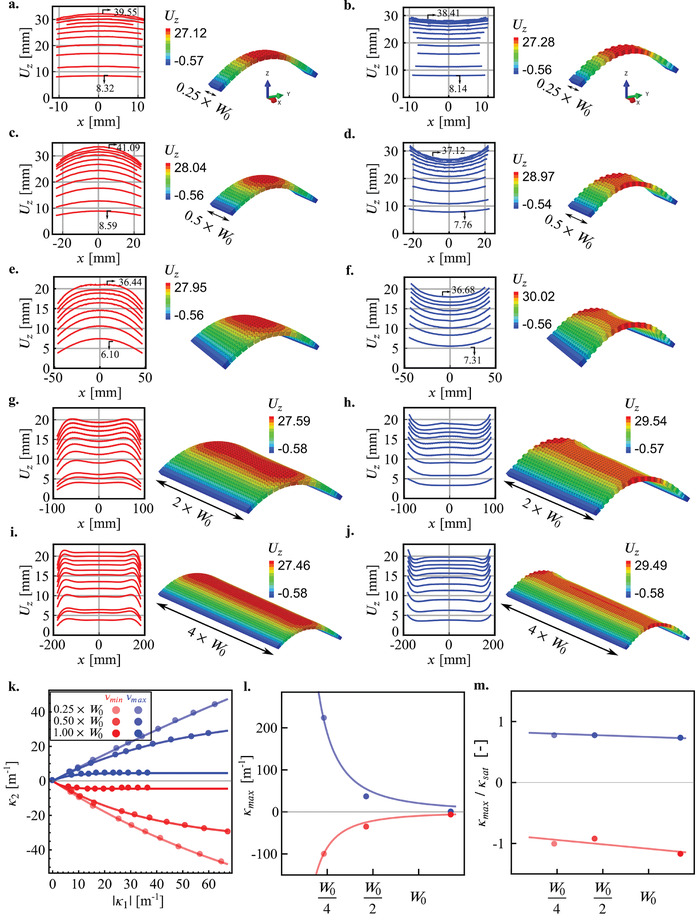
The effects of the width ($W_{0}$ = 92 mm) of the meta‐plates on their shape under compression for two extreme cases with the a,c,e,g,i) negative and b,d,f,h,j) positive values of the Poisson's ratios. The values of $\kappa_{1}$ in m^−1^ are shown on different profiles in the left insets of (a–f) (see Movies S1–S4, Supporting Information). The other parameters were maintained constant (h = 5 mm). The insets in these sub‐figures show the displacement distribution in the Z direction under equal induced curvature (i.e., $\kappa_{1}$ ≈ 20 m^−1^). k–m). The insets show the parameters of the exponential fit for $\kappa_{2}=\kappa_{max}\;\times \;\left[1-e^{\frac{-\kappa_{1}}{\kappa_{sat}}}\right]$.

To study the effects of the boundary conditions on the second principal curvature, we created computational models of meta‐plates with smaller and larger widths (i.e., W/4, W/2, 2W, and 4W) than that of our reference models (i.e., W) for both the maximum and minimum values of the Poisson's ratio. For those cases, we kept the out‐of‐plane thickness of the meta‐plates (i.e., h = 5 mm) constant. We find the second principal curvature to be highly dependent on the width of the specimens for both auxetic and non‐auxetic meta‐plates (**Figures** **3**k,l). As the width of the meta‐plates increases, the absolute value of $\kappa_{2}$ at the center of the meta‐plates decreases (Figures 3a–j). For sufficiently large widths (i.e., plate's width >2W), the middle part of the meta‐plates exhibits a near‐zero value of $\kappa_{2}$ (Figures 3g–j). Closer to the boundaries, however, curvatures similar to what was observed for the reference models are observed (Figures 3g–j). An important observation is that when the width of the plate is much larger than the length, curvatures are always localized in the free boundaries with a specific penetration depth.

Global bending in the transverse direction is only observed for meta‐plates with relatively small widths (i.e., plate's width < W) (Figure 3). As in the classical case of isotropic plates,^[^ ^76^ ^]^ the bending of wide meta‐plates is limited to their edges. Nevertheless, in contrast with classical plates, this maximum width is much larger than the typical limit ${\left(h/\kappa_{1}\right)}^{1/2}$. For instance, in the specimens shown in Figure 3, we observe uniform transverse bending up to a width of 92 mm (Figure 3e,f), for an applied curvature equal to $\kappa_{1}$ = 36 m^−1^. In the case of a plain material, the limit in width for a uniform transverse bending under the same applied curvature is on the order of ${\left(h/\kappa_{1}\right)}^{1/2}\approx 12\;mm$.

A comparison of the deformation of plain (i.e., non‐architected) plates with equivalent isotropic elastic properties with those of meta‐plates showed similar non‐linear behaviors for the negative and positive values of the Poisson's ratio. Both types of plates (i.e., plain and meta‐plates) were linearly deformed and the transverse induced curvature saturated by imposing the longitudinal curvature. The plain plates, however, cannot predict the maximum curvature achieved by the meta‐plates. For an equivalent set of geometrical parameters, the results obtained with plain plates cannot be directly extrapolated to meta‐plates and the induced curvature is significantly larger in the case of meta‐plates (Figure S1, Supporting Information).

**Figure 4:**
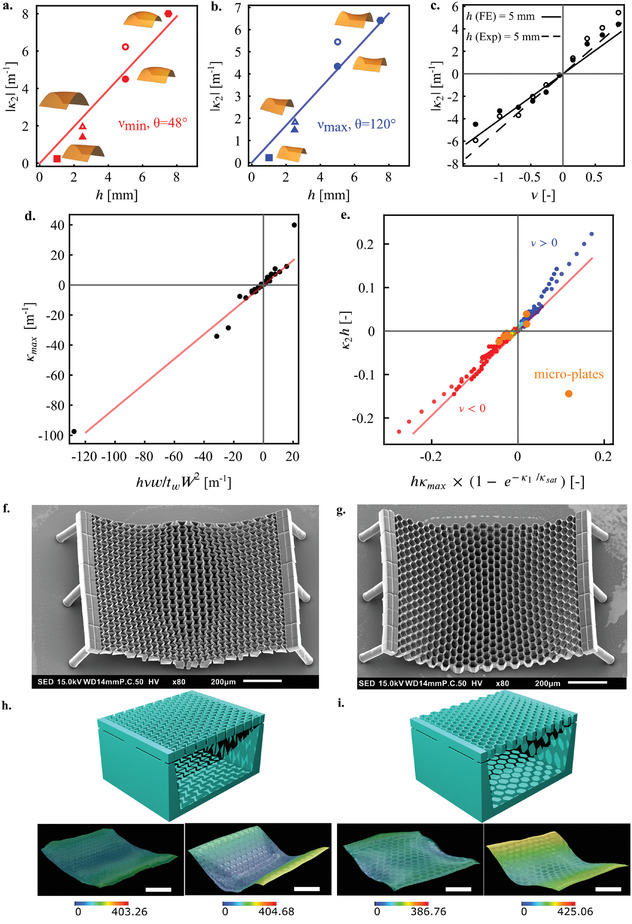
a,b) Change in the absolute value of the second principal curvature with respect to the thickness of the plate for the most positive (a) and most negative (b) values of the Poisson's ratios, respectively corresponding to θ = 120° (ν = 0.87) and 48° (ν = −1.35). The other parameters were maintained constant ($\kappa_{1}\approx$ 20 m^−1^, $W_{0}$ = 92 mm). The filled and unfilled markers respeticely denote the numerical and experimental data points. The lines in (a) and (b) are fits to the numerical results. c) The evolution of $\kappa_{2}$ as a function of the Poisson's ratio for the plates with h = 5 mm ($\kappa_{1}$ and W were kept constant). $\kappa_{2}$ exhibits a linear relationship with ν, where the values of the coefficient, m, are listed in Table S2, Supporting Information. d) The relationship between the maximum curvature and the geometrical parameters of the meta‐plate in Equation (3). e) The dimensionless relationship between both principal curvatures in the form $\kappa_{2}=\kappa_{max}\;\times \;\left[1-e^{\frac{-\kappa_{1}}{\kappa_{sat}}}\right]$). f,g) The SEM images of the 3D printed microscale meta‐plates. These micro‐meta‐plates are deformed by capillary forces during solvent evaporation. h,i) The 3D design of the microscale meta‐plates. The insets show the dynamic deformation of the microscale meta‐plates when interacting with ethanol (see Movies S5–S6, Supporting Information). The white scale bars in the subfigures represent 200 µm.

The linear dependence of the transverse curvature on the thickness of the meta‐plate and the Poisson's ratio is confirmed experimentally. In **Figure** **4**a–c, experimental measurements are compared with FE simulations for an imposed curvature (i.e., $\kappa_{1}\approx$ 20 m^−1^). There is a linear relationship between the thickness of the meta‐plate, h, and its second principal curvature, $\kappa_{2}$, (i.e., $\kappa_{2}\propto h$) obtained using our computational models for extremely negative (Figure 4a) and extremely positive (Figure 4b) values of the Poisson's ratio. This is similar to the linear dependency observed in beams.^[^ ^76^ ^]^ Our experimental observations show a clear linear relationship between the second principal curvature and the Poisson's ratio (i.e., $\kappa_{2}\propto \nu$) as expected (Figure 4c). As the thickness of the meta‐plate increases, the proportionality constant between the second principal curvature and the Poisson's ratio increases (Figure S2 and Table S2, Supporting Information). Furthermore, regardless of the value of the Poisson's ratio, the second principal curvature linearly decreases as the in‐plane thickness of the struts of the meta‐plates increases (Figure S2, Supporting Information).

From the above‐mentioned analyses, we propose the following dimensionless scaling relationship between the second principal curvature and the other geometrical parameters of the meta‐plate (Figures 4d,e).

(3) $$\begin{array}{c} \kappa_{2}\;=\;\kappa_{\max}\left(1-e^{\frac{-\kappa_{1}}{\kappa_{\text{sat}}}}\right)\;,\;\kappa_{\max}\;=\;\frac{\nu hw}{t_{w}W^{2}},\;\frac{\kappa_{\max}}{\kappa_{\text{sat}}}\;=\;\alpha \nu \end{array}$$

Here, we found α = 0.02. This low value may be interpreted as a consequence of the anisotropy of the meta‐plate. Using this expression, we can readily tailor the intrinsic curvature of the meta‐plates simply by adjusting the corresponding geometrical parameters of the plates. We can then use this prediction to design the micro‐architecture of the meta‐plates.

To show the length scale independence of the proposed empirical equation between two principal curvatures, we scaled down our reference meta‐plates and fabricated auxetic and non‐auxetic meta‐plates at the microscale using a submicron 3D printing technique (i.e., two‐photon polymerization). The microscale meta‐plates were made of a resin (i.e., IP‐Q, see Experimental Section). The addition and subsequent evaporation of ethanol induced deformations in the micro‐plates as a result of a capillary force exerted during the solvent evaporation (Figures 2, 4). This leads to dynamic changes in the curvature of the top surface of the micro‐plates (Figures 4h,i, bottom row subfigures, and Movies S5–S6, Supporting Information). We quantified the level of the induced curvatures through optical microscopy. After the application of the scaling law (Figure 4e), the $\kappa_{2}$ values of the micro‐plates were in the range of those determined for the macro‐plates, confirming that the proposed relationship (Equation (3)) captures the essential physics of the problem across multiple length scales.

**Figure 5:**
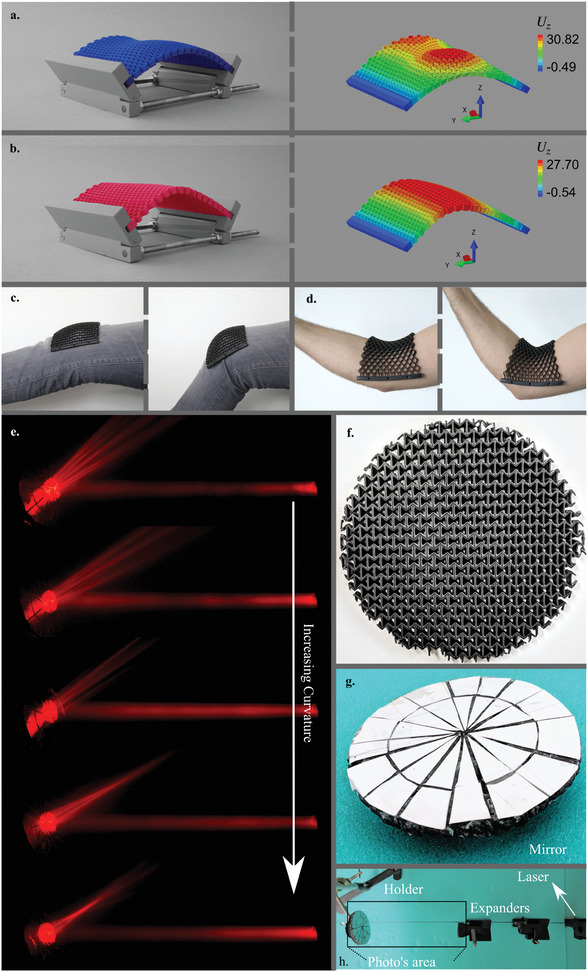
a) Complex curvatures can result from a combination of auxetic and non‐auxetic unit cells. Two examples are presented here where the meta‐plate was divided into two equal regions of which one was auxetic and the other non‐auxetic. b) Meta‐plates with a functional gradient in their Poisson's ratio from negative to positive values. The position of the maximum curvature in the meta‐plate changed as a result (the color code in the right subfigures (a,b) show the displacements in the *Z*‐direction). c,d) A demonstration of potential soft wearable devices, especially at the joints, where double curvatures (c,d) are required to create a shape‐fitting curvature. e–h) Adaptive mirrors (e) were made by covering auxetic meta‐plates (f) with a highly reflective aluminum foil (g). e,h) The adaptive mirrors were then exposed to laser beams. e) The focused laser beams reflected off the surface show flat, concave, or convex mirrors.

We have shown that, in contrast to plain plates (i.e., a plate with negligible thickness) whose Gaussian curvature under compression is invariably zero, meta‐plates could be used to fabricate surfaces with a wide range of positive or negative Gaussian curvature. This approach provides a route for the rational design of thin‐walled engineering structures with a wide range of applications and complex curvature requirements. As an illustration, a meta‐plate divided into regions with positive and negative values of the Poisson's ratio could be used to create spatial variations in the Gaussian curvature from positive to negative values (**Figure** **5**a). A meta‐plate with a gradient of the Poisson's ratio (gradual variations from positive to negative values) allows for adjusting the location of the maximum curvature as well as for modulating the shape of the curved surface (Figure 5b). Many other design approaches where regions with different thicknesses and/or Poisson's ratios are combined to meet complex curvature requirements can be envisioned as well. This could, for example, be used in soft wearable robotics (e.g., exoskeleton^[^ ^83^, ^84^ ^]^) that need to morph the curved contours that define the shape of the human body (Figures 5c,d). With a simple tuning of the geometrical parameters of the elementary cells, a wide range of curvatures could also be exploited to create adaptive optical devices (e.g., mirrors) whose focal length is dependent on the level of the applied compressive load (Figures 5e–h).

## 3 Conclusion

The meta‐plates proposed here combine the advantages of thin‐walled structures, such as efficiency in load transfer and low weight with those of intrinsically curved designs, such as adaptive shape morphing features and ease of interaction with the human body. Ultimately, the possibility to start from an initially flat shape translates into additional benefits, including the opportunity to incorporate complex surface‐related functionalities, including nanopatterns and flexible electronics into the final 3D device.^[^ ^85^ ^]^ The relation we found between the different physical length scales remains so far empirical. We hope our study will motivate additional research that provides a deeper physical ground to the scaling law we obtained and enables its extension to nonlinear regimes. Nevertheless, this simple law offers a simple basis for programming cellular meta‐plates and achieving complex 3D structures that hold great promise for practical applications.

## 4 Experimental Section

The macroscale specimens and their grippers from poly(lactic acid) (PLA) filaments (MakerPoint PLA 750 gr Natural) were additively manufactured using a fused deposition modeling 3D printer (Ultimaker 2+). The experimental setup was designed such that a fixed level of longitudinal deformation (i.e., δL = 30 mm) was applied to the upper side of the specimens while fixing the lower sides. The hinge joint at the boundaries of the fixture allowed both ends of the meta‐plates to rotate freely and to create an out‐of‐plane deformation under compression (Figures 1c,e). The size of the unit cells (i.e., 2c/w=3) and the overall size of the meta‐plates (i.e., $W_{0}$ = 92 mm, $L_{0}$ = 130 mm) were kept constant for all the reference designs. The wall thickness, $t_{w}$, of the struts making up the unit cells was 0.51 mm. The only variable, therefore, was the angle of the unit cells, θ, which determined the Poisson's ratio of the meta‐plate. All dimensions of the meta‐plates are presented in Figure 1 and Table S1, Supporting Information. 10 specimens with θ angles varying between 48° and 120° were fabricated to cover a wide range of Poisson's ratios (Table S1, Supporting Information). Although the thickness, h, of the meta‐plates was set to 5 mm, two additional specimens with lower out‐of‐plane thicknesses (i.e., h = 2.5 and 1 mm) were also fabricated using the maximum and minimum values of the θ angle (i.e., the most negative and most positive values of the Poisson's ratio).

The outer contour of the deformed structures was captured by a 3D scanner (Scan‐In‐A‐Box, FX, ASUS mini beamer, resolution of both cameras: 1280 × 800 pixels). The specimens were photographed from at least eight different angles. The images were then rigidly registered using the software accompanying the 3D scanner (IDEA). After noise removal, the point clouds were imported into CloudCompare software (V.2.9.1) for further analysis.

FE calculations were conducted with Abaqus (Dassault Simulia, V6.14). After importing the geometry of the specimens, a linear brick element (C3D8R, Abaqus) was used for the simulations. A linear elastic material model was used for PLA (E = 3.5 GPa, ν= 0.3). Two reference points were placed on the top and bottom of the meta‐plates. These points were kinematically coupled with the corresponding upper and lower nodes lying on the surface of the specimens. To perform the buckling analysis, a unit concentrated force was applied to the upper reference node. The upper and lower sides of the structures were set free to rotate perpendicularly to the applied loading direction while their other degrees of freedom were constrained. Linear buckling analysis was then performed using the eigenvalue solver available in Abaqus. The displacements of the nodes corresponding to the first buckling mode were then introduced as geometrical imperfections to perform the nonlinear post‐buckling analyses.

A compressive displacement equal to 16 mm was set in the computational models so as to achieve a similar out‐of‐plane deformation as observed in the experiments, after registering the deformations obtained from the post‐buckling analyses to those of the experimental data (Figures 1c–f). The displacements in the out‐of‐plane direction (i.e., Z‐direction, $U_{z}$) of the top surfaces of the computational models were extracted as point clouds. The numerical and experimental point clouds were then registered in CloudCompare (V.2.9.1). The first, $\kappa_{1}$, and second, $\kappa_{2}$, principal curvatures were defined based on Equation (1) to the data points at the center point of the meta‐plates (Figures 1d,f). A sphere‐fitting algorithm available in Mathematica (version 11.3, Wolfram Research, US) was used for that purpose. Four additional plate thicknesses (i.e., h= 1, 2.5, 5, 7.5 mm) were also considered in the computational simulations to evaluate the effects of the thickness of the meta‐plates on their curvatures. For the meta‐plates with a thickness of 5 mm, 22 additional simulations with varying in‐plane thicknesses (i.e., $\frac{t_{w}}{4},\frac{2t_{w}}{4},\frac{3t_{w}}{4},\frac{5t_{w}}{4}$), widths (i.e., $\frac{W}{4},\frac{W}{2},2W,4W$) and unit cell sizes (i.e., 75%, 150%, 300% of w while maintaining 2c/w=3) were performed for the cases with the most negative and positive values of the Poisson's ratio.

Microscale meta‐plates were fabricated with a two‐photon polymerization 3D printer (Photonic Professional GT machine, Nanoscribe, Germany). A laser power of 100% and a scanning speed of 100 000 µm s^−1^ were applied to print the structures in the DiLL configuration using a 10× objective. A droplet of the IP‐Q resin (Nanoscribe) was placed on a silicon substrate and was exposed to a femtosecond infrared laser beam (wavelength = 780 nm) to fabricate the designed structures. The samples were then developed in propylene glycol monomethyl ether acetate (from Sigma Aldrich) for 25 min and were dried at room temperature. Furthermore, a scanning electron microscope (SEM, JSM IT100, JEOL) was used to acquire high‐resolution images after gold‐sputtering (JFC‐1300, JEOL, Japan) of the dried specimens (Figures 4f,g). The dynamic curvature of the micro‐structures was evaluated in air and liquid (ethanol) (Sigma Aldrich) through an analysis of optical microscopy images (Keyence Digital Microscope VHX‐6000) (Figures 4h,i).

Soft meta‐plates (Figures 5c,d) were additively manufactured using a polyjet 3D printer technique (Objet350 Connex3 3D printer, Stratasys, USA) that works on the basis of inkjet‐deposited droplets of a photopolymer followed by curing under ultraviolet light. A commercially available hyperelastic polymer (i.e., Agilus30 Black, FLX985, Stratasys, USA) was used for the fabrication of these specimens.

To create the adaptive mirrors (Figures 5e–h), the top surface of a meta‐plate with the most negative value of the Poisson's ratio was covered by aluminum foils with a high degree of reflectivity (Figures 5f,g). The specimen was then placed in a holder and was subjected to the laser beams passed from expanders (Figure 5h). The laser beams were used to demonstrate the change in the focal point and curvature of the mirror as a function of the deformation.

## Conflict of Interest

The authors declare no conflict of interest.

## Author Contributions

M.J.M. and A.G. contributed equally to this work. M.H. and A.A.Z. jointly supervised this work. M.J.M., M.H., and A.A.Z. designed the research. M.J.M., K.N., and S.J. performed numerical simulations. M.J.M. and K.N. performed experiments (3D scan). M.J.M., N.T., and S.J.P.C. performed images analysis and data collection from numerical simulations and 3D scans. A.G., J.B., and M.H. developed the theoretical models. M.N.‐G. and A.A. helped with 3D micro‐fabrication. M.J.M. and M.N.‐G. performed SEM and microscopy imaging. M.J.M., A.G., J.B., M.H., and A.A.Z. performed the data analysis and interpretation. M.J.M. and A.G. performed data visualizations. A.G. designed and performed the adaptive mirror experiment. M.J.M., J.B., M.H., and A.A.Z. wrote the manuscript. All the authors critically revised the manuscript for its intellectual content and approved the manuscript.

## Supporting information

Supporting Information

Supplemental Movie 1

Supplemental Movie 2

Supplemental Movie 3

Supplemental Movie 4

Supplemental Movie 5

Supplemental Movie 6

## Data Availability

Research data are not shared.
