# Supplementary material for: Curvature Induced by Deflection in Thick Meta‐Plates
Source: Adv Mater. 2021 Jun 13;33(30):2008082. doi: 10.1002/adma.202008082 (PMC11469285; doi:10.1002/adma.202008082)
Supplement: Supplementary file 1 — Supporting Information [file ADMA-33-2008082-s001.pdf]

© 2021 Wiley-VCH GmbH

ADVANCED MATERIALS

# Supporting Information

for *Adv. Mater.,* DOI: 10.1002/adma.202008082

# Curvature Induced by Deflection in Thick Meta-Plates

*Mohammad J. Mirzaali,* Aref Ghorbani, Kenichi Nakatani, Mahdiyeh Nouri-Goushki, Nazli Tümer, Sebastien J. P. Callens, Shahram Janbaz, Angelo Accardo, José Bico, Mehdi Habibi, and Amir A. Zadpoor*

# Curvature induced by deflection in thick meta-plates

M. J. Mirzaali[a,*,1], A. Ghorbani[b,1], K. Nakatani[a], M. Nouri-Goushki[a], N. Tümer[a], S. J. P. Callens[a], S. Janbaz[a], A. Accardo[d], J. Bico[c], M. Habibi[b,2], A. A. Zadpoor[a,2]

[a] *Department of Biomechanical Engineering, Faculty of Mechanical, Maritime, and Materials Engineering, Delft University of Technology (TU Delft), Mekelweg 2, 2628 CD, Delft, The Netherlands*
[b] *Physics and Physical Chemistry of Foods, Department of Agrotechnology and Food Sciences, Wageningen University, Wageningen, The Netherlands*
[c] *Sorbonne Université, Université Paris Diderot and Laboratoire de Physique et de Mécanique des Milieux Hétérogenes (PMMH), CNRS, ESPCI Paris, PSL Research University - 10 rue Vauquelin, 75005 Paris, France*
[d] *Department of Precision and Microsystems Engineering, Delft University of Technology, Mekelweg 2, 2628 CD Delft, The Netherlands*

**Table S1.** The geometrical parameters and Poisson's ratio of the specimens tested in this study. The parameters are defined in Figures 1c and 1e.

| $\theta$ [°] | $\varphi$ [°] $= 90 - \theta$ | $a$ [$mm$] | $b$ [$mm$] | $\upsilon_{yx}$ [$-$] | $\upsilon_{xy}$ [$-$] |
|---|---|---|---|---|---|
| 48 | -42 | 12.00 | 6.73 | -1.35 | -0.74 |
| 56 | -34 | 10.57 | 6.03 | -0.97 | -1.03 |
| 64 | -26 | 9.52 | 5.56 | -0.69 | -1.45 |
| 72 | -18 | 8.71 | 5.26 | -0.46 | -2.17 |
| 80 | -10 | 8.04 | 5.08 | -0.25 | -3.96 |
| 88 | -2 | 7.45 | 5.00 | -0.05 | -19.67 |
| 96 | 6 | 6.87 | 5.03 | 0.16 | 6.44 |
| 104 | 14 | 6.25 | 5.15 | 0.37 | 2.67 |
| 112 | 22 | 5.52 | 5.39 | 0.61 | 1.64 |
| 120 | 30 | 4.63 | 5.77 | 0.87 | 1.15 |

**Table S2.** The parameters of the linear regression fit ($\kappa_2 = m \times \upsilon$) for meta-plates with different out-of-plane thickness, $h$, values (Figure S1a).

| $h$ [$mm$] | $m$ [$1/m$] |
|---|---|
| 1 | 0.2 |
| 2.5 | 1.4 |
| 5 | 4.2 |
| 7.5 | 6.7 |
| 5 (EXP) | 5 |

[*] Corresponding author. Tel.: +31-15-2783133
*E-mail address:* m.j.mirzaali@tudelft.nl
[1] These authors contributed equally to this work.
[2] These authors jointly supervised this work.

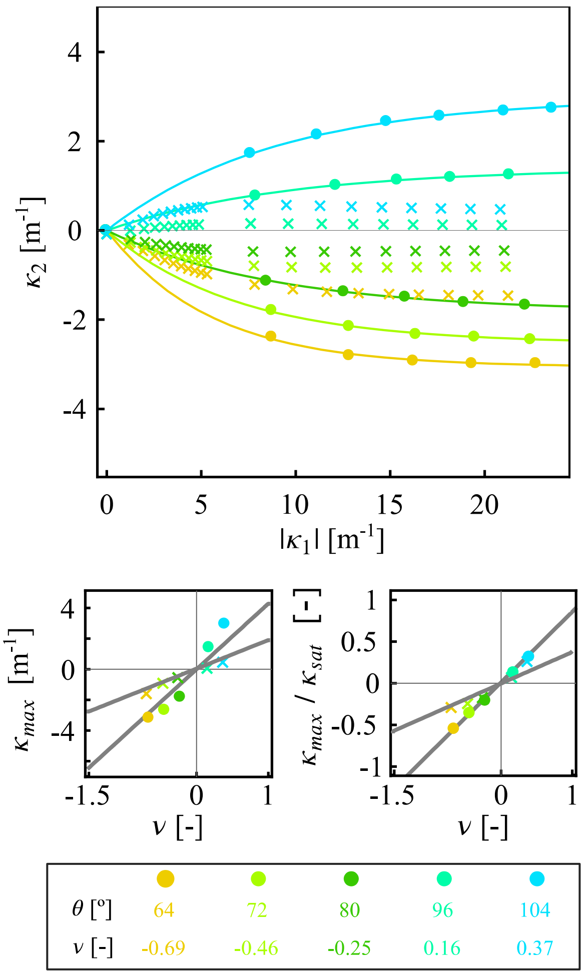


**Figure S1.** Induced curvature, $\kappa_1$, *vs.* imposed curvature, $\kappa_2$, calculated for meta-plates (circular markers) and plain (*i.e.*, non-architected) plates with equivalent elastic properties (crossed markers).

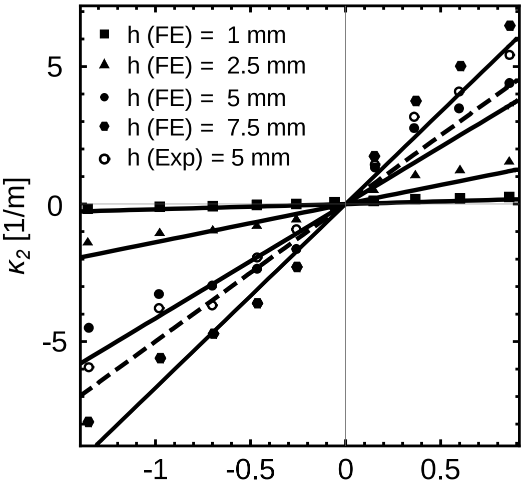


**Figure S2.** The evolution of $\kappa_2$ as a function of the Poisson's ratio for plates with different values of the thicknesses, $h$, ($\kappa_1$, $W$ are kept constant). $\kappa_2$ exhibits a linear relationship with $\upsilon$, where the coefficient, $m$, is presented in Table S2 (supplementary document) for each plate thickness.
